# Rho-inhibiting C2IN-C3 fusion toxin inhibits chemotactic recruitment of human monocytes ex vivo and in mice in vivo

**DOI:** 10.1007/s00204-017-2058-y

**Published:** 2017-09-18

**Authors:** Tobias Martin, Amelie Möglich, Ina Felix, Christina Förtsch, Anne Rittlinger, Annette Palmer, Stephanie Denk, Julian Schneider, Lena Notbohm, Mona Vogel, Hartmut Geiger, Stephan Paschke, Markus Huber-Lang, Holger Barth

**Affiliations:** 1 Institute of Pharmacology and Toxicology, University of Ulm Medical Center, Albert-Einstein-Allee 11, 89081 Ulm, Germany; 2 Institute of Clinical and Experimental Trauma-Immunology, University of Ulm Medical Center, Helmholtzstrasse 8/2, 89081 Ulm, Germany; 3 Department of General and Visceral Surgery, University of Ulm Medical Center, Albert-Einstein-Allee 23, 89081 Ulm, Germany; 4 Institute of Molecular Medicine, University of Ulm, Ulm, Germany; 5 Division of Experimental Hematology and Cancer Biology, CCHMC, Cincinnati, USA

**Keywords:** Trauma, Inflammation, Monocytes, Chemotaxis, Rho, Clostridial C3 toxin

## Abstract

Bacterial protein toxins became valuable molecular tools for the targeted modulation of cell functions in experimental pharmacology and attractive therapeutics because of their potent and specific mode of action in human cells. C2IN-C3lim, a recombinant fusion toxin (~50 kDa) of the Rho-inhibiting C3lim from *Clostridium* (*C.*) *limosum* and a non-toxic portion of the *C. botulinum* C2 toxin (C2IN), is selectively internalized into the cytosol of monocytic cells where C3lim specifically ADP-ribosylates Rho A and -B, thereby inhibiting Rho-mediated signaling. Thus, we hypothesized that these unique features make C2IN-C3lim an attractive molecule for the targeted pharmacological down-regulation of Rho-mediated functions in monocytes. The analysis of the actin structure and the Rho ADP-ribosylation status implied that C2IN-C3lim entered the cytosol of primary human monocytes from healthy donors ex vivo within 1 h. Moreover, it inhibited the fMLP-induced chemotaxis of human monocytes in a Boyden chamber model ex vivo. Similarly, in a 3-dimensional ex vivo model of extravasation, single cell analysis revealed that C2IN-C3lim-treated cells were not able to move. In a clinically relevant mouse model of blunt chest trauma, the local application of C2IN-C3lim into the lungs after thorax trauma prevented the trauma-induced recruitment of monocytes into the lungs in vivo. Thus, C2IN-C3lim might be an attractive lead compound for novel pharmacological strategies to avoid the cellular damage response caused by monocytes in damaged tissue after trauma and during systemic inflammation. The results suggest that the pathophysiological role of clostridial C3 toxins might be a down-modulation of the innate immune system.

**Electronic supplementary material:**

The online version of this article (doi:10.1007/s00204-017-2058-y) contains supplementary material, which is available to authorized users.

## Introduction

Recently we and others identified monocytes/macrophages as target cells for the C3 protein toxins (~25 kDa) from *Clostridium* (*C.*) *botulinum* (C3bot1) and *C. limosum* (C3lim) (Fahrer et al. 2010; Dmochewitz et al. 2013; Christow et al. 2013; Rotsch et al. 2012; Tautzenberger et al. 2013; Rohrbeck et al. 2014). The clostridial C3 toxins catalyze the covalent transfer of an ADP-ribose moiety from NAD onto Asn-41 of the GTPases Rho A, -B, -C (Aktories and Frevert 1987; Aktories et al. 1995; Just et al. 1992; Han et al. 2001; for review see Aktories 2011), which inhibits the Rho-mediated signal transduction in mammalian cells (Rubin et al. 1988; Chardin et al. 1989; Etienne-Manneville and Hall 2002). Moreover, C3 toxins are the only known specific Rho-inhibitors. ADP-ribosylated Rho-GDP is trapped as Rho-GDI complexes in the cytosol and, therefore, is not able to translocate to the cytoplasmic membrane to become activated into Rho-GTP (Genth et al. 2003). As a consequence, the subsequent activation of the Rho-effector molecules is inhibited. Rho A is a key regulator of the actin cytoskeleton, thereby controlling actin-dependent processes including cell adhesion and migration. Therefore, the inhibition of Rho-signaling, i.e. by C3 toxin, results in reorganization of the actin cytoskeleton associated with an altered cell morphology and the Rho/actin-mediated cellular processes are inhibited (for review see Vogelsgesang et al. 2007).

The clostridial C3 proteins do not cause cytotoxic effects in other tested cell types. Epithelial cells or fibroblasts respond to C3 toxin-treatment with a rearrangement of their actin cytoskeleton and morphological changes only after long incubation periods (>24 h) with high C3 toxin concentrations (>10 µg/mL). Therefore, it was suggested that C3 proteins are taken up by non-specific pinocytosis (Chardin et al. 1989; Wiegers et al. 1991; Verschueren et al. 1997; for review see Vogelsgesang et al. 2007; Barth et al. 2015). Thus, the role of clostridial C3 toxins as virulence factors remains unknown. However, C3-treatment of leukocytes prevents migration, adhesion, and phagocytosis, which suggests a potential pathophysiological role of C3 toxins towards these cells of the innate immune system (Worthylake et al. 2001; Laudanna et al. 1996; Liu et al. 2002; Caron and Hall 1998; Aepfelbacher et al. 1996; Wheeler and Ridley 2007).

Both C3lim and C3bot1 are efficiently taken up into the cytosol of cultured monocytes and macrophages, when incubated with comparatively low C3 toxin concentrations (<1 µg/mL) for short periods (<3 h) (Fahrer et al. 2010; Rohrbeck et al. 2015). The reason for the efficient uptake of the C3 toxins into the cytosol of these immune cells is not understood. However, Rohrbeck et al. reported that C3bot1 binds to proteinaceous structures on J774A.1 macrophages and that these cells exhibit significantly more C3-binding sites than other cell types (Rohrbeck et al. 2014). Moreover, vimentin has been identified as a C3-binding protein involved in C3 uptake (Adolf et al. 2016). The C3-catalyzed ADP-ribosylation of Rho results in characteristic morphological changes of the intoxicated macrophages, due to the reorganization of the actin cytoskeleton, and inhibits essential macrophage functions in vitro such as migration or phagocytosis (Aepfelbacher et al. 1996; Fahrer et al. 2010; Caron and Hall 1998). Previous results suggest that the clostridial C3 toxins are internalized into monocytes/macrophages via an endocytic mechanism comparable to bacterial AB-type protein toxins, because their uptake into the host cell cytosol was reduced when the endosomal acidification was prevented (Fahrer et al. 2010; Dmochewitz et al. 2013). Interestingly, a recombinant C3lim fusion toxin (C2IN-C3lim) showed significantly higher cytotoxic activity towards cultured macrophage-like cells in vitro compared to C3bot1 and C3lim (Tautzenberger et al. 2013). This is most likely due to C2IN (~25 kDa), the enzymatically inactive membrane translocation domain of the *C. botulinum* enzyme C2I that enhances the intracellular protein transport across endosomal membranes (Barth et al. 1998, 1999). Noteworthy, C2IN had no effect on the enzyme activity of C3lim and C2IN-C3lim did not affect other cell types (Barth et al. 1998, 1999, 2002; Tautzenberger et al. 2013; Gacanin et al. 2017).

Here, we investigated the intoxication of primary human monocytes from healthy blood donors by C3bot1, C3lim and C2IN-C3lim ex vivo. Because C2IN-C3lim was the most efficient C3 toxin, its inhibitory effect on chemotaxis of primary human monocytes ex vivo was investigated in more detail that included a 3-dimensional model of extravasation. Moreover, the recruitment of monocytic cells from the blood into the lungs after blunt chest trauma in mice was investigated in vivo (Knoeferl et al. 2003). Taken together, the results suggest an immunosuppressive mode of action of C3 toxins and identified the recombinant fusion toxin C2IN-C3lim as an attractive candidate for the targeted pharmacological down-modulation of monocyte recruitment and accumulation in damaged tissues/organs which might be detrimental after traumatic injury.

## Materials and methods

### Materials

Cell culture media (DMEM, RPMI) and fetal calf serum were purchased from Invitrogen (Karlsruhe, Germany) and cell culture materials from TPP (Trasadingen, Switzerland) and Sarstedt (Nümbrecht, Germany). Penicillin–streptomycin, Page Ruler prestained Protein ladder, and goat-anti-mouse-Alexa488 antibody were purchased from Thermo Fisher Scientific (Ulm, Germany). Complete^®^ protease inhibitor and streptavidin-peroxidase were from Roche (Mannheim, Germany) and biotinylated NAD^+^ from R&D Systems GmbH (Wiesbaden-Nordenstadt, Germany). Thrombin was purchased from Amersham Biosciences Europe GmbH (Freiburg, Germany), the nitrocellulose blotting membrane from Whatman^®^ (Dassel, Germany), and the enhanced chemiluminescence (ECL) system from Millipore (Schwalbach, Germany). Monoclonal anti-ß-actin antibody (clone AC-15, mouse) was from Sigma-Aldrich (Hamburg, Germany).

### Expression and purification of recombinant proteins

The proteins C3bot1, C3lim, C2I, C2IIa, and C2IN-C3lim were over-expressed as recombinant glutathione S-transferase-fusion proteins in *E. coli* as described earlier (Barth et al. 1998, 1999; Fahrer et al. 2010). C3bot1, C3lim, C2I, and C2IIa were expressed in BL21, C2IN-C3lim was also expressed in the *E. coli* strain Rosetta. After over-expression, the toxins were purified by affinity chromatography. In brief, *E. coli* (BL21 or Rosetta) harboring the plasmids pGex-C3bot1, pGex-C3lim, pGex-2T-C2I, pGex-C2II or pGex-C2IN-C3lim were grown at 37 °C in Luria–Bertani (LB) medium supplemented with 100 µg/mL ampicillin to an optical density of 0.6–0.8. To induce protein expression, isopropyl-ß-d-thiogalactopyranoside (IPTG) was added to a final concentration of 0.2 mM and the cultures incubated at 29 °C for 12 h. The bacteria were harvested by centrifugation for 10 min at 4 °C at 5400*g*, resuspended in lysis buffer (10 mM NaCl, 20 mM Tris, pH 7.4) containing 1% Triton X-100 and 1% PMSF, and disrupted by sonification. Cellular debris was sedimented for 10 min at 4 °C at 20,300*g* and the supernatant was incubated with 500 µL/L culture Glutathione-Agarose 4B beads (Macherey–Nagel, Düren, Germany) at 4 °C for 12 h. The suspension was centrifuged for 10 min at 4 °C at 1000*g*, the beads were washed two times with washing buffer (150 mM NaCl, 20 mM Tris–HCl, pH 7,4) and one time with PBS, and finally the immobilized protein was incubated with thrombin (10–20 NIH units/L culture) for 1 h at room temperature to remove the GST-tag. The protein-containing supernatant was obtained by centrifugation for 30 s at 4 °C at 10,600*g* and for the elimination of thrombin the supernantant was incubated with 30–100 µL/L culture Benzamidin Sepharose beads (GE Healthcare, Uppsala, Sweden) for 10 min at room temperature.

The purified proteins were subjected to 12.5% sodium dodecyl sulfate–polyacrylamide gel electrophoresis (SDS-PAGE) and stained with Coomassie blue, and the protein concentration was determined via densitometry by using ImageJ (NIH, Bethesda, USA).

### Removal of endotoxin from recombinant protein

Endotoxin was removed from purified C2IN-C3lim using the EndoTrap^®^ HD column (Hyglos, Bernried, Germany). The C2IN-C3lim-solution was enriched with 0.1 mM CaCl_2_. The depletion column was regenerated with the provided regeneration buffer and equilibrated with PBS + 0.1 mM CaCl_2_. The C2IN-C3lim-solution was applied to the column and after complete pass through of the C2IN-C3lim suspension, the remaining toxin was eluted with PBS + 0.1 mM CaCl_2_. Examination of endotoxin-removal was performed on the C2IN-C3lim suspension after EndoTrap^®^ HD-treatment using the Hycult^®^ biotech limulus amebocyte lysate chromogenic endpoint assay-kit (Hycultec, Beutelsbach, Germany) according to the manufacturer’s instructions.

### Cell culture and intoxication assays

J774A.1 macrophage-like cells (from DSMZ, Braunschweig, Germany) were cultured at 37 °C and 5% CO_2_ in DMEM medium, containing 10% heat-inactivated fetal calf serum (FCS) and 1% penicillin–streptomycin. Cells were routinely scraped off and reseeded three times per week. For intoxication experiments, cells were seeded in 24-well culture dishes. After incubation for the indicated periods, C3bot1, C3lim, or C2IN-C3lim were applied and the cells were incubated at 37 °C and 5% CO_2_. For control, cells were incubated without toxin. To analyze the morphological changes caused by the toxins, the cells were visualized by using a Zeiss Axiovert 40 CFL microscope (Oberkochen, Germany) with a Jenoptik progress C10 CCD camera (Jena, Germany). The number of intoxicated cells was determined by cell-counting using ImageJ (NIH, Bethesda, USA) as described earlier (Fahrer et al. 2010).

### Migration assay (scratch test)

J774A.1 macrophage-like cells were grown to confluence in 12-well plates; subsequently, one defined scratch per well was induced using a pipette tip to remove a portion of the cells. Cells were treated with C2 toxin (C2I + C2IIa), C3bot1, or C2IN-C3lim in DMEM medium containing 1% penicillin–streptomycin, first for 3 h without serum to enable a more efficient uptake of the toxins. 10% heat-inactivated FCS was then added and cells incubated at 37 °C and 5% CO_2_ for further 21 h. For control, cells were incubated in the same medium without toxin. The wells were visualized directly after scratch-treatment and toxin-application (0 h), and after 24 h of incubation, by using a Zeiss Axiovert 40 CFL microscope (Oberkochen, Germany) with a Jenoptik progress C10 CCD camera (Jena, Germany).

### Isolation of primary human monocytes

Peripheral blood monocytes of healthy volunteers (ethics votum 51/14 of the Local Independent Ethics Committee of the University of Ulm) were isolated using OptiPrep™ density gradient-medium following the application sheet C10 (Axis Shield, Oslo, Norway). The blood, collected using S-Monovette^®^ 7.5 mL K3E (Sarstedt, Nümbrecht, Germany) and Safety-Multifly^®^ 21 Gtube200 mm (Sarstedt, Nümbrecht, Germany), as well as all solutions and equipment were cooled down to 4 °C.

Two milliliter of 54% iodixanol solution (5.4 vol. OptiPrep™-medium and 0.6 vol. of 8.5% (w/v) NaCl) was mixed with 10 mL of whole blood by several very gentle inversions, and a centrifugation medium was created by mixing 12.6 vol. OptiPrep™-medium with 47.7 vol. RPMI 1640 + 10% FCS + 1% penicillin–streptomycin. Four milliliter diluted blood was pipetted into a 15-mL test tube (Corning Science Mexico, Reynosa, Mexico), after which 5 mL gradient medium was added carefully and finally 0.5 mL RPMI 1640 + 10% FCS + 1% penicillin–streptomycin was pipetted on top (in order to avoid banding of the cells at a liquid/air interface). During the following centrifugation (700*g*, 30 min, 4 °C, no break during deceleration), the monocytes float to the top of the gradient layer. After collection, the cells were gently diluted with 2 vol. of 8.5% NaCl and centrifuged for 10 min at 400*g* at 4 °C with no break during deceleration. The harvested cells were gently suspended in RPMI 1640 + 10% FCS + 1% penicillin–streptomycin. The fraction of monocytes exceeded 90% as confirmed by Pappenheim-stained cytospin preparations.

### Intoxication of primary human monocytes and sequential ADP-ribosylation of Rho

The primary human monocytes were counted directly after isolation, transferred to 12-well plates, and incubated overnight at 37 °C with 5% CO_2_. For toxin treatment, the respective toxins were pipetted directly into the medium and cells were incubated at 37 °C with 5% CO_2_. Next, cells were scraped from the surface, flushed by pipetting, and transferred to 1.5 mL reaction tubes. After short centrifugation, pellet and supernatant were separated.

To analyze the ADP-ribosylation status of Rho from primary human monocytes, the cells were lysed in 30 µL of a ribosylation buffer containing 20 mM Tris–HCl (pH 7.5), 1 mM EDTA, 1 mM DTT, 5 mM MgCl_2_, complete^®^ protease inhibitor; 23 µL of this cell lysate was then incubated for 30 min at 37 °C with biotin-labeled NAD^+^ (10 µM) and 300 ng of C3bot1 (25 µL total volume). The reaction was stopped by adding 5 × SDS-sample buffer (625 mM Tris/HCl pH 6.8, 20% SDS, 8.5% glycerol, 0.2% bromophenol blue, 100 mM DTT) and heating for 10 min at 95 °C. Subsequently, the ADP-ribosylated (i.e. biotin-labelled) proteins were analyzed by Western blotting with streptavidin peroxidase and enhanced chemiluminescence reaction as described earlier (Fahrer et al. 2010).

### SDS-PAGE and Western Blotting

For immunoblot analysis, equal amounts of protein were separated by SDS-PAGE according to the method of Laemmli (Laemmli 1970). Afterwards, the proteins were transferred to a nitrocellulose membrane which was then blocked for 1 h at RT, or alternatively overnight at 4 °C, with 5% dry milk powder in PBS containing 0.1% Tween-20 (PBS-T). For detection of the biotin-labeled Rho, the samples were probed with streptavidin-peroxidase for 1 h followed by washing steps with PBS-T. Thereafter, the biotin-labeled, i.e. bition-ADP-ribosylated Rho was visualized using the ECL system according to the manufacturer’s instructions as described earlier (Fahrer et al. 2010). For comparison of the samples, the intensity of the bands showing the biotin-labeled Rho was evaluated via densitometry by using the Adobe Photoshop software (version 7.0, Adobe Systems GmbH, Munich, Germany) as described earlier (Schuster et al. 2017). Ponceau S staining of the membrane and Coomassie staining of the gel were used to confirm comparable amounts of protein.

### Immunofluorescence microscopy

Primary human monocytes were incubated in µ-slide 8-well plates (ibidi, Planegg/Martinsried, Germany) over night. The cells were then incubated with C2IN-C3lim for 3 h at 37 °C in 5% CO_2_ or left untreated for control. Cells were fixed with 4% paraformaldehyde (PFA) for 20 min at room temperature, permeabilized with 0.4% Triton X-100 for 5 min at room temperature, treated with 100 mM glycine for 2 min at room temperature, and blocked with 5% dry milk powder in PBS-T for 30 min at 37 °C. Actin was stained with mouse-anti-ß-actin antibody (1:2000 in 5% dry milk powder in PBS-T) for 30 min at 37 °C and Alexa488-conjugated anti mouse antibody (1:2000 in 5% dry milk powder in PBS-T) for 30 min at 37 °C. After three washing steps with PBS-T, the nuclei were counterstained with Hoechst 33342 (1:10,000 in 5% dry milk powder in PBS-T) for 5 min at 37 °C. Finally, the samples were analyzed by immunofluorescence microscopy using a LSM 710 laser scanning confocal microscope system, equipped with a 63× oil immersion objective (Zeiss, Germany) coupled to an XL-LSM 710 S incubator. Images were processed and merged with ImageJ software (NIH, Bethesda, USA).

### Chemotaxis assay (Boyden chamber test)

Isolated primary human monocytes (in RPMI1640 + 10% FCS + 1% penicillin–streptomycin) were incubated for 40 min with C2IN-C3lim at 37 °C. For control, cells were left untreated or treated with the actin depolymerizing *C. botulinum* C2 toxin. For labeling, 2′,7′-Bis-(2-carboxyethyl)-5-(and -6)-carboxyfluorescein (BCECF, Molecular Probes, Eugene, USA) was applied to the cells and cells, then incubated in the dark for an additional 20 min. After centrifugation at 340*g* at room temperature for 5 min, cells were suspended in HBSS + Ca^2+^ + 0.1% BSA and loaded into the upper chambers of a 96-well chemotaxis mini-chamber (Neuroprobe, Gaithersburg, USA). The lower chambers were loaded with either fMLP (100 nM) in HBSS + Ca^2+^ + 0.1% BSA or HBSS + Ca^2+^ + 0.1% BSA without fMLP and separated from the upper one by a polycarbonate membrane of 8 µm porosity (Neuroprobe, Gaithersburg, USA). The cells were incubated in the mini-chamber for 40 min at 37 °C in the dark. After incubation, the membrane was removed, washed with DPBS + 0.1% BSA, and dried in the dark. To determine the chemotactic activity of the samples, the amount of cells that had migrated into the polycarbonate membrane was measured by cytofluorometry (Cytofluor II, PerSeptive Biosystems, Cambridge, USA) at 485/20 nm and 530/35 nm. Samples were measured at a minimum of *n* = 5 replicates.

### 3-dimensional extravasation assay

To identify whether a reduced migration towards, or through, the pores of the Boyden chamber membrane was responsible for the reduction in migrated cell numbers in response to toxin treatment, we measured migration speed on 2D surfaces and in 5 × 5 µm wide 3D microchannels simulating the membrane pores.

Isolated primary human monocytes (in RPMI1640) were incubated for 1 h with C2IN-C3lim (2 μg/mL), C2IN-C3lim (1 μg/mL) + C2IIa (2 μg/mL), C2I (1 μg/mL) + C2IIa (2 μg/mL) at 37 °C. Control cells were left untreated. The microfluidic setup consisted of a two-chambered glass chip (iX-factory, Dortmund, Germany) with one inlet and outlet per chamber, connected to microfluidic tubing (IDEX, Lake Forest, USA) and a syringe pump (KDS 200, KD Scientific, Holliston, USA). The chip was placed in an onstage incubator (Incubator S, PeCon, Erbach, Germany) on an inverted microscope (TS100, Nikon, Tokyo, Japan) equipped with a CFI S P-Fluor ELWD ADM 20×/0.45 objective (Nikon, Tokio, Japan) and camera (Orca 05, Hamamatsu, Hamamatsu City, Japan). Temperature was kept stable at 37 °C. After flushing the microfluidic system and chip with medium (RPMI1640), the left chamber was loaded with treated or untreated human monocytes (3 × 10^6^ cells/mL). The right chamber was loaded with fMLP (100 nM in RPMI1640). For time lapse imaging, bright-field pictures were acquired for 1 h every 5 s using HCImage 2.4 (Hamamatsu, Hamamatsu City, Japan). Cell tracking was performed using image data processing and analysis code written in MATLAB (The MathWorks, Natick, USA). Cell paths were then evaluated in relation to these regions (cell velocity before and in channel, target focus towards other chamber, time required to enter channel).

### Blunt chest trauma mouse model

Male C57BL/6 J mice (Jackson Laboratories, Sulzfeld, Germany; Janvier Labs, Le Genest-Saint-Isle, France), 7–12 weeks old (body weight 22–28 g) were used to induce a blunt chest trauma (Knoeferl et al. 2003). In the mouse husbandry a day–night cycle of 12 h, a mean room temperature of 21 °C, and a mean humidity of 50–60% was applied. Animals were allowed free access to water and food (Altromin 1314 Forti, Altromin, Lage-Lippe, Germany). Animal work and specimen collection were reviewed and approved by the institutional review board/ethics committee of the University of Ulm and the Federal Animal Care and Use Committee (No. 1185 and 1321, Regierungspraesidium Tuebingen, Germany). Mice were anesthetized with sevoflurane (3.5 vol%), fixed on an acrylic glass plate in supine position, and chest and abdomen were shaved. Blunt chest trauma was induced by a single blast wave centerd on the thorax of the mice leading to a bilateral lung contusion, a soft tissue damage without any bone injuries (Knoeferl et al. 2003; Liener et al. 2003). Immediately and 8 h after trauma, phosphate-buffered saline (PBS) or C2IN-C3lim was instilled intratracheally. For preparation and isolation of the bronchoalveolar lavage (BAL) fluid and alveolar macrophages (AM), 24 h after injury, the lung was flushed once with 600 µL cold phosphate-buffered saline (PBS; Gibco, New York, USA) to collect BAL fluid and afterwards another four times with 600 µL for AM isolation (BAL 2). After centrifugation of BAL fluids (400*g,* 10 min at 4 °C), supernatants of BAL 1 were removed and stored at −80 °C until mediator analysis. For cell counting the pellets of BAL 1 and BAL 2 were resuspended in PBS, pooled, stained with crystal violet, and counted in the Neubauer chamber.

### Analysis of monocyte cell numbers in lung after TXT and sham treatment

12-week-old C57BL76 J mice (Janvier Labs, Le Genest-Saint-Isle, France) were used for TXT induction or sham treatment. Mice were killed 2 or 24 h later using Sevoflurane. Thereafter, cardiac puncture on the open thorax was performed to isolate blood. Left atrium was nicked and lungs were perfused with PBS (3–5 mL) via the right ventricle to remove leftover blood. Mouse lungs were dissected into single lobes, all external tissue was removed, and lungs were washed in PBS. To obtain single cells, lung dissociation kit (Miltenyi Biotec, Bergisch Gladbach, Germany) was used according to manufacturer’s protocol. Subsequently, red cell lysis was performed, cells were counted, and 10^6^ cells/sample stained as described in Yu et al. (2016) with slight modifications. Shortly, FC-block (eBioscience, Frankfurt, Germany) was added for 15 min, and then the following Abs were added for 1 h: eFluor-450 Anti-mouse Ly-6G, clone RB6-8C5 (eBioscience), V500 Rat-anti-mouse CD45, clone 30-F11 (BD Bioscience, Heidelberg, Germany), AlexaFluor700 Anti-mouse CD11b, clone M1/70 (eBioscience), Pe-eFluor610 Anti-mouse MHC II(I-A/I-E), clone M5/114.15.2 (eBioscience), AlexaFluor780 Anti-mouse Ly6C, clone HK1.4 (eBioscience), PerCP-Cy5.5 Anti-mouse CD24, clone M1/69 (eBioscience), APC-Anti-mouse CD64, clone X54-5/7.1 (Biolegend, Koblenz, Germany), Pe-Cy7 Anti-mouse CD11c, clone N418 (eBioscience), and Propidium iodide solution (Sigma-Aldrich, Hamburg, Germany) were added before measuring the samples on a BD LSR II flow cytometer. Compensation was performed before each experiment. Absolute cell number was calculated as the product of whole cell number/single events acquired in flow cytometry × monocytes measured in flow cytometry. Relative percentage of Monocytes was calculated using FACS Diva 8.0 software. Monocytes as CD45^+^, CD11b^+^, CD64^+^, Ly6G^−^ and MHC II^−.^

## Results

### The recombinant C2IN-C3lim fusion toxin inhibits the migration of macrophages in vitro

**Fig. 1:**
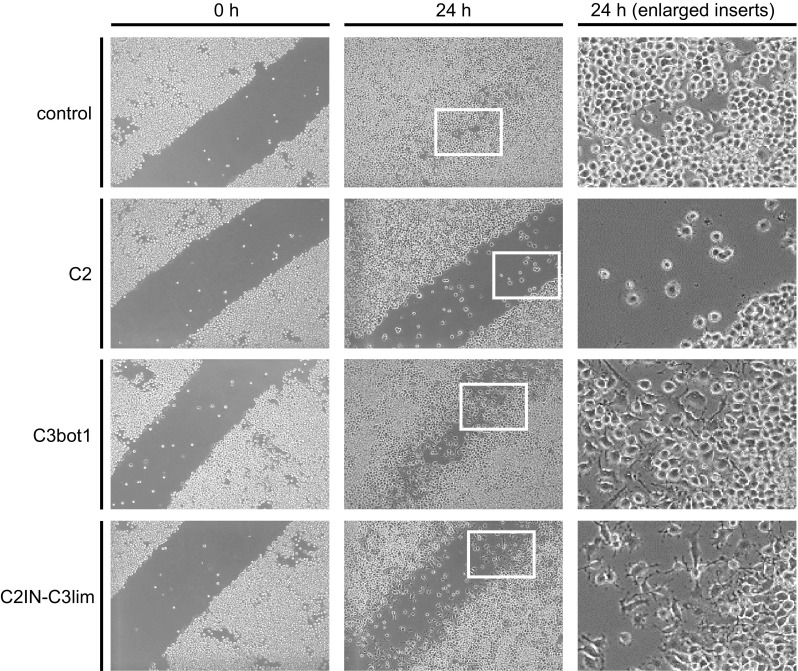
The recombinant C2IN-C3lim fusion toxin most efficiently inhibits the migration of J774A.1 macrophages in vitro. J774A.1 cells were grown near to confluence and a scratch with a pipet tip was made into the monolayer. Pictures from the cells were taken (indicated as 0 h) and cells were grown for further 24 h at 37 °C in the absence or presence of the following toxins: C2I + C2IIa (1 + 2 µg/mL), C2IN-C3lim (300 nM), C3bot1 (300 nM). After application of the toxins, the cells were incubated for 3 h at 37 °C without serum to enable a more efficient uptake of the toxins into the cells. Then, 10% FCS was added to the medium and cells were further incubated in the presence of serum. After 24 h of incubation, pictures from the cells were taken (indicated as 24 h). The white inserts from the middle panel are shown as enlarged pictures in the right panel to demonstrate the changes in cell morphology after an 24 h treatment with C3bot1 and C2IN-C3lim

**Fig. 2:**
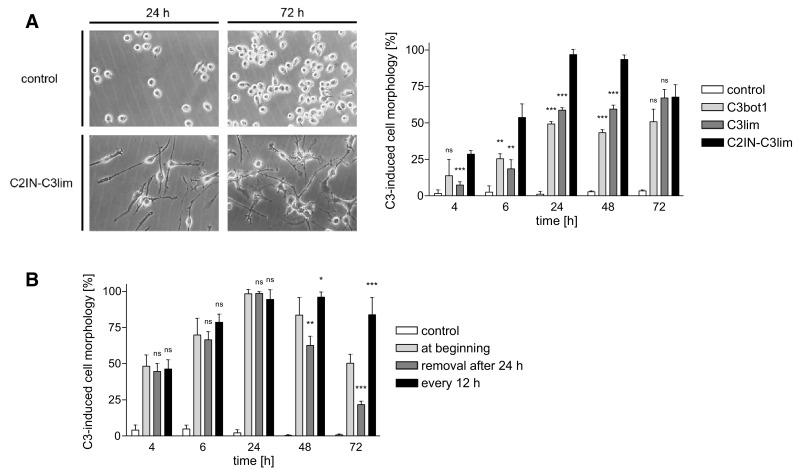
The recombinant C2IN-C3lim fusion toxin most efficiently intoxicates J774A.1 macrophages in vitro. **a** J774A.1 cells were incubated for 72 h in the presence of either C3bot1, C3lim or C2IN-C3lim (each 1 µg/mL), or left untreated for control. After the indicated incubation periods pictures were taken as shown in the left panel exemplarily for untreated and C2IN-C3lim-treated cells. The amount of cells showing the characteristic C3-induced cell morphology was determined from the pictures (right panel). Values are given as mean ± SD (*n* = 3) and significance was tested between “C2IN-C3lim” vs. “C3bot1” and “C2IN-C3lim” vs. “C3lim” using Student’s *t* test. (*ns* not significant, **p < 0.01, ***p < 0.001). **b** For each group, except the control, C2IN-C3lim (1 µg/mL) was added into the medium and J774A.1 cells were incubated at 37 °C. In the first sample, C2IN-C3lim was applied only at the beginning. In the second group, C2IN-C3lim was applied at the beginning but removed after 24 h by exchange of the medium and the cells were incubated for further 48 h without C2IN-C3lim. In the third group, C2IN-C3lim was applied at the beginning and additionally at every 12 h by an exchange of the toxin-containing medium. For control, cells were left untreated. In each case, pictures from the cells were taken and the amount of cells showing C3-induced cell morphology was determined. Values are given as mean ± SD (*n* = 3) and significance was tested between “treatment at beginning” vs. “removal of medium after 24 h” and between “treatment at beginning” vs. “addition of C2IN-C3lim every 12 h” using Student’s *t* test (ns, not significant, *p < 0.05, **p < 0.01, ***p < 0.001)

First, the most potent C3 toxin for the subsequent ex vivo and in vivo experiments was identified. Based on the results that C3bot1 down-modulates the migration of macrophages in vitro (Wheeler and Ridley 2007; Rotsch et al. 2012) and that recombinant C2IN-C3lim acts significantly stronger on RAW macrophages and derived osteoclasts than the natural C3bot1 (Tautzenberger et al. 2013), we investigated whether C2IN-C3lim is also more potent regarding the inhibition of macrophage migration. To this end, confluently grown J774A.1 macrophages were subjected to a scratch where cells were removed by a scraping with pipet tip and cells were further incubated in the absence or presence of either C3bot1 or C2IN-C3lim. For negative control, cells were incubated without any toxin and for positive control with the actin-inhibiting *C. botulinum* C2 toxin. The scratch was mainly overgrown in the untreated cells after 24 h of incubation but remained widely open when the cells were treated with C2 toxin because this toxin inhibits migration (Fig. 1). C3bot1 also prevented the migration of cells into the scratch area but the inhibitory effect was obviously stronger for C2IN-C3lim (Fig. 1), indicating that this recombinant Rho-inhibitor intoxicates the macrophages more efficiently. As shown in the enlarged pictures from the white inserts (right panel), the cells treated with C3bot1 or C2IN-C3lim showed the characteristic C3 toxin-induced changes of their morphology with branched protrusions and this effect was also stronger after treatment with C2IN-C3lim. Indeed, the effect of C2IN-C3lim on the morphology of J774A.1 cells was stronger compared to C3bot1 and C3lim (Fig. 2a). Because the enzyme activity among the three C3 proteins used in this experiment is comparable, these results strongly suggest that more C3 enzyme reached the cytosol of the macrophages when C2IN-C3lim was used instead of a mere C3 protein and that the C2IN portion enhances the delivery of C3lim into the cytosol.

However, the result from Fig. 2a suggests that the intoxication of the macrophages by C2IN-C3lim was transient and some cells recovered after 48 and more obviously after 72 h, in contrast to C3bot1- and C3lim-treated cells. To investigate this effect in more detail, J774A.1 cells were incubated with C2IN-C3lim under different conditions (Fig. 2b). In one approach, the cells were treated with a single dose of C2IN-C3lim exactly as in Fig. 2a. In a parallel approach, C2IN-C3lim was added at the beginning and additionally after every 12 h. In the third approach, a single dose C2IN-C3lim was added to the medium at the beginning but removed from the medium after 24 h and the cells were then further incubated without C2IN-C3lim. While cells strongly recovered after removal of the toxin, they remained intoxicated after repeated application of C2IN-C3lim (Fig. 2b). This result demonstrates the transient and reversible intoxication of macrophages by recombinant C2IN-C3lim, which we observed earlier for epithelial cells and astrocytes when we delivered C2IN-C3lim by a separate transporter protein into the cytosol of these cells (Barth et al. 1998, 1999).

Thus, C2IN-C3lim is not only most potent among the C3 toxins, but also the only C3 toxin which acts reversibly in cells, suggesting that its mode of action can be better controlled and may be reversed after over-dosage. Taken together, the recombinant C2IN-C3lim should be the optimal C3 Rho-inhibitor for pharmacological applications to down-modulate monocyte migration/chemotaxis in vivo and was, therefore, used in all subsequent experiments.

### C2IN-C3lim ADP-ribosylates Rho and thereby rearranges the actin cytoskeleton in primary human monocytes ex vivo

**Fig. 3:**
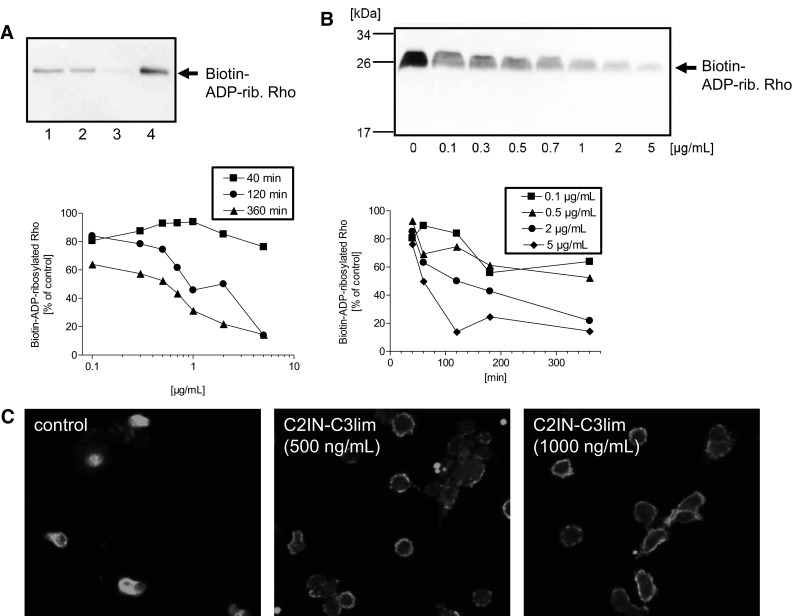
C2IN-C3lim ADP-ribosylates Rho and thereby rearranges the actin cytoskeleton in primary human monocytes ex vivo. **a** Primary human monocytes were isolated from healthy donors and incubated ex vivo in medium for 3 h at 37 °C with 500 ng/mL C2IN-C3lim (lane 1), 1 µg/mL C2IN-C3lim (lane 2), C2IN-C3lim (500 ng/mL) + C2IIa (1 µg/mL) (lane 3) or without toxin for control (lane 4). The cells were lysed and the ADP-ribosylation status of Rho from these cells (2.8 × 10^5^ cells per sample) analyzed by sequential ADP-ribosylation with biotin-NAD^+^ as co-substrate and Western blotting. Note: A strong signal of biotin-ADP-ribosylated Rho in the Western blot indicates that Rho was not ADP-ribosylated in the intact cells while a weak signal of biotin-ADP-ribosylated Rho means that most of the Rho was already ADP-ribosylated in the living cells during incubation with the respective toxin, and therefore did not serve as substrate for the subsequent in vitro ADP-ribosylation reaction. **b** Concentration- and time-dependent effects of C2IN-C3lim on primary human monocytes. Analysis of the ADP-ribosylation status of Rho from monocytes, which were incubated for different times with increasing concentrations of C2IN-C3lim in the medium. The ADP-ribosylation status of Rho from the cells (4 × 10^5^ cells per sample) was analyzed as described before and the Western blot shows the result for the 6 h incubation period. For comparison of the samples, the intensity of biotin-labeled Rho was evaluated via densitometry. The graphs show the intensity of biotin-ADP-ribosylated Rho, which corresponds to the amount of cellular Rho that was not ADP-ribosylated in the living cells during the incubation with C2IN-C3lim. **c** Primary human monocytes were isolated from healthy donors and incubated at 37 °C for 3 h in the presence of C2IN-C3lim (500 ng/mL or 1000 ng/mL). For control, cells were left untreated. Cells were fixed and actin stained with an antibody against ß-actin. The stained actin was visualized by confocal fluorescence microscopy

Having identified C2IN-C3lim as the most potent C3 toxin for macrophage cell lines, we next confirmed that it also intoxicates primary human monocytes. Monocytes were isolated from peripheral blood from healthy donors. Success of isolation was confirmed by Pappenheim staining. The monocytes (2.8 × 10^5^ cells per sample) were incubated for 3 h with 0.5 or 1 µg/mL of C2IN-C3lim. For negative control, cells were left untreated. For positive control, cells were incubated with C2IN-C3lim in combination with the separate transporter protein C2IIa, which delivers C2IN-C3lim into the cytosol via the C2 toxin uptake pathway. After the indicated incubation periods, the cells were lysed and the ADP-ribosylation status of Rho analyzed by sequential ADP-ribosylation and Western blotting. In this assay, a strong signal of ADP-ribosylated Rho, as observed for the untreated control cells, indicated that the total amount of Rho served as substrate during the in vitro ADP-ribosylation reaction with biotinylated NAD as co-substrate. Therefore, Rho protein was ADP-ribosylated in vitro and detected as biotinylated Rho in the Western blot. In contrast, only a negligible signal was detected when the cells were treated with C2IN-C3lim plus C2IIa transporter. Most of the Rho protein was already ADP-ribosylated in the living cells and, therefore, did not serve as substrate for the subsequent in vitro ADP-ribosylation with the biotinylated NAD (Fig. 3a). As shown in Fig. 3a, the signal of the biotinylated Rho from cells treated with C2IN-C3lim alone was weaker compared to the signal obtained from control cells. Although this effect was weaker compared to the effect caused after delivery of C2IN-C3lim via the C2IIa transporter, the result implicates that C3 enzyme activity reached the cytosol of primary human monocytes when C2IN-C3lim alone was applied, because the ADP-ribosylation of Rho occurs in the host cell cytosol and, therefore, represents a sensitive and specific endpoint to detect cytosolic C3 enzyme activity.

To analyze the time- and concentration-dependent effects of C2IN-C3lim on primary human monocytes in more detail, the cells were incubated for up to 6 h with increasing concentrations of C2IN-C3lim in the medium, and the ADP-ribosylation status of Rho from these cells (4 × 10^5^ cells per sample) was analyzed by sequential ADP-ribosylation as described in detail before. The results shown in Fig. 3b clearly indicate that the amount of biotin-ADP-ribosylated Rho decreased in a time- and concentration-dependent manner. This means that less non-ADP-ribosylated Rho was present in the samples from these cells due to the increasing C3 enzyme activity in their cytosol, implicating that the toxin reached the cytosol of primary human monocytes in a time- and concentration-dependent manner with first detectable effects on Rho after about 1 h of incubation.

To confirm that the C3-catalyzed ADP-ribosylation of Rho indeed results in the expected down-stream effects on the actin cytoskeleton, the actin of the monocytes was stained with a specific antibody against ß-actin after 3 h of incubation with C2IN-C3lim. As shown in Fig. 3c, the actin was homogenously distributed in untreated control cells but only a faint ring-shaped signal of actin was detectable in the periphery of cells treated with C2IN-C3lim, indicating the redistribution of the actin cytoskeleton after C3-induced inhibition of Rho-signaling in the monocytes.

### C2IN-C3lim inhibits chemotaxis and 3-dimensional extravasation of primary human monocytes ex vivo

**Fig. 4:**
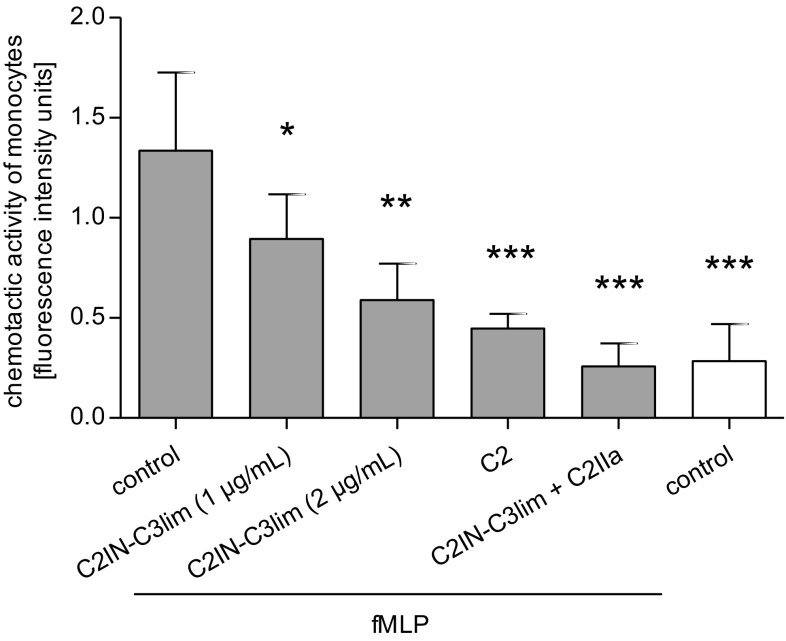
C2IN-C3lim inhibits chemotaxis of primary human monocytes ex vivo. Primary human monocytes were isolated from healthy donors and 1.6 × 10^5^ cells per sample were incubated at 37 °C for 1 h in RPMI medium without FCS with or without the following toxins: C2I (1 µg/mL) + C2IIa (2 µg/mL), C2IN-C3lim (1 µg/mL) + C2IIa (2 µg/mL), C2IN-C3lim (1 µg/mL) and C2IN-C3lim (2 µg/mL). During the last 20 min of incubation, the fluorescence dye BCECF was added into the medium to label the cells. Subsequently, cells were applied into the upper wells of the trans-well Boyden chamber and incubated for 40 min towards the chemoattractant fMLP (100 nM) in the lower chamber wells. For control, cells were not exposed to fMLP. The membrane filters between the upper and lower chambers had a pore size of 8 µM. Chemotaxis was monitored by measuring the fluorescence signal of the cells associated with the filter after a 40 min incubation period. Values are given as mean ± SD (*n* = 6) and significance was tested for each sample against the “fMLP-treated control group” using Student’s *t* test (*p < 0.05, **p < 0.01, ***p < 0.001)

Since treatment with C2IN-C3lim rapidly results in Rho-modification and rearrangement of the actin cytoskeleton in primary human monocytes, the effect of this Rho-inhibitor on chemotaxis of these cells was investigated. Primary human monocytes were labeled with a fluorescent dye and pretreated with increasing concentrations of C2IN-C3lim for 1 h. For negative control, cells were left untreated. For positive control, cells were treated with the actin depolymerizing C2 toxin. The cells were subjected into the upper reservoir of a trans-well chamber that contained fMLP as chemoattractant in the lower chamber. Both chambers were separated by a filter and the amount of cells that migrated towards the fMLP was determined by fluorescence measurement of the cell-containing filters. As shown in Fig. 4, less cells migrated towards the fMLP after treatment with C2IN-C3lim and this inhibition was concentration-dependent. Although the inhibitory effect of C2 toxin was stronger, the data clearly show that treatment with C2IN-C3lim decreases the chemotactic migration of primary human monocytes ex vivo. However, from this analysis, it remained unclear if the monocytes do not recognize the chemoattractant due to Rho-inhibition, or do not migrate, or both.

**Fig. 5:**
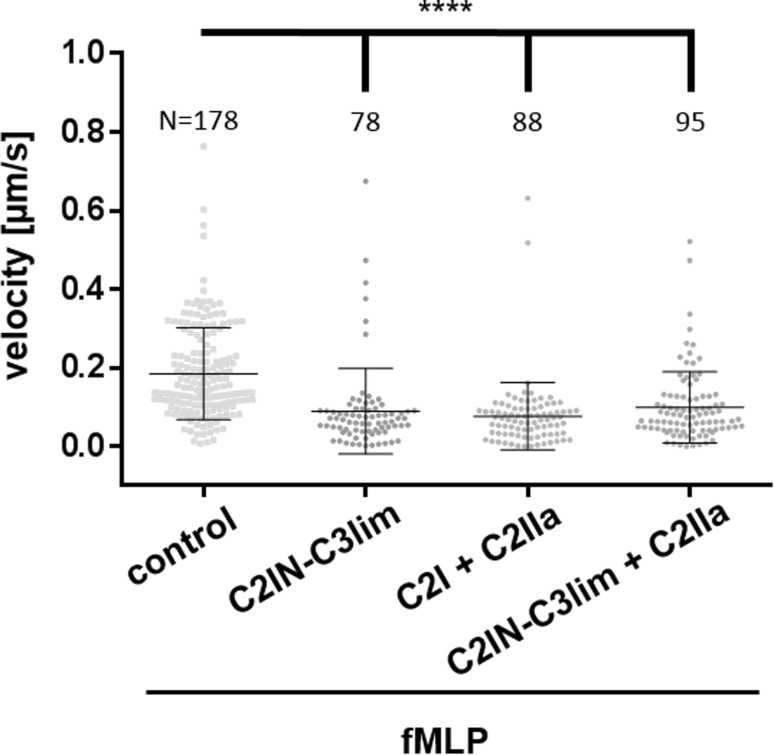
C2IN-C3lim inhibits the 3-dimensional extravasation of primary human monocytes ex vivo. Primary human monocytes were isolated from healthy donors and 3 × 10^6^ cells per sample were incubated at 37 °C for 1 h in RPMI1640 medium without FCS with or without C2IN-C3lim (2 µg/mL), C2I (1 µg/mL) + C2IIa (2 µg/mL) and C2IN-C3lim (1 µg/mL) + C2IIa (2 µg/mL). The cells were applied into the left chamber and exposed towards the chemoattractant fMLP (100 nM) from the right chamber. The chambers were connected by seven 5 × 5 µm wide and 100 µm long channels. Chemotaxis was investigated by microscopic monitoring of cells every 5 s for 1 h and subsequent cell tracking was performed to determine cell velocity (also see supplement movie S1 A and B). The massive dots in each group represent single measured cells. In each group the measurements from five independent preparations were pooled. For each group the total cell number is indicated. Values are given as mean ± SD. Significance was tested using Kruskal–Wallis test with Dunn’s multiple comparisons test (****p < 0.0001)

Therefore, the effect of C2IN-C3lim on the chemotactic trans-migration of primary human monocytes through 3-dimensional channels that mimic the situation in tissues during the extravasation of leukocytes from blood vessels was investigated (Fig. 5). The data presented in Fig. 5 are from individual preparations. The massive dots in each group represent single measured cells. In each group, the measurements from five independent preparations were pooled. From the single cell-based time-lapse analysis which is shown in the movies in the supplementary materials, it became obvious that the monocytes do not migrate at all after treatment with the Rho-inhibitor C2IN-C3lim. This result suggested that treatment with C2IN-C3lim should prevent the chemotactic recruitment of monocytes from the blood into damaged or inflamed tissue areas.

### C2IN-C3lim inhibits the recruitment of monocytic cells into the lungs after murine blunt chest trauma in vivo

**Fig. 6:**
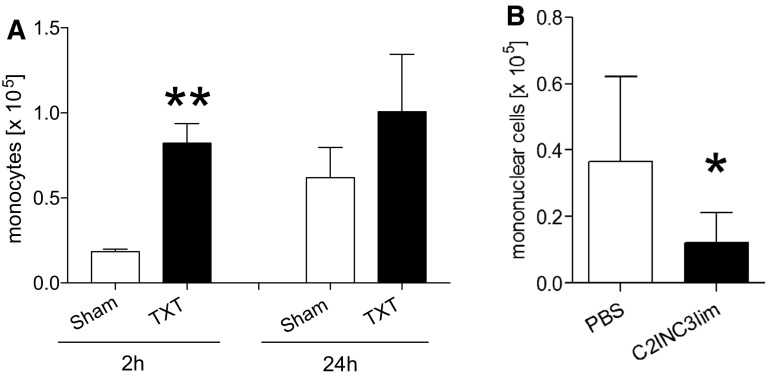
C2IN-C3lim decreases the recruitment of monocytic cells into the lungs after blunt chest trauma (TXT) of mice in vivo. **a** Absolute number of monocytes in blood-depleted lungs of mice at 2 and 24 h after sham- or TXT-treatment of the mice. Absolute monocyte number was calculated as the product of whole cell count after lung dissociation/single events measured in flow cytometry * monocytes number in flow cytometry. Monocytes were stained in accordance with Yu et al. (2016) (columns represent mean ± 1 SEM, *n* = 4 (2 h); *n* = 6 (24 h), student’s *t* test; **p ≤ 0.01).** b** In 7–8 weeks old male C57BL/6 J mice, TXT was induced. Immediately, and 8 h after TXT, phosphate-buffered saline (100 µL PBS) for control or 100 µL C2IN-C3lim (i.e. 1 µg C2IN-C3lim) were instilled intratracheally. 24 h later, bronchoalveolar lavage fluids (BAL) were taken and monocytic cells were counted in a Neubauer chamber after crystal violet staining. Values are given as mean ± SD (*n* = 8 in each group) and significance was tested using Student’s *t* test; *p < 0.05 vs. TXT + PBS

As reported in earlier studies (Knoeferl et al. 2003) and shown for 2 h (significant) and 24 h (trend) after TXT in Fig. 6a, there is an increased amount of monocytes in the lungs of mice after blunt thorax trauma (TXT) compared to sham-treated mice, due to the enhanced chemotactic recruitment of these cells from the blood after TXT (Knoeferl et al. 2003). To prove the hypothesis that C2IN-C3lim-treatment prevents the chemotactic recruitment of monocytes from the blood into the alveolar space after TXT, mice were subjected to a single blast wave to induce a TXT and then C2IN-C3lim was applied into the trachea (i.t.) of these animals directly after and 8 h after blast wave treatment. For control, PBS as vehicle was applied i.t. instead of C2IN-C3lim. After 24 h the broncho-alveolar lavage fluids of the lungs were collected, the cells were specifically stained, and the content of monocytic cells was determined. All mice survived the treatment within the 24 h and no adverse effects of the C2IN-C3lim treatment were observed. Importantly, in mice treated with C2IN-C3lim after TXT the amount of monocytes was significantly reduced (Fig. 6b), indicating that C2IN-C3lim-treatment after trauma prevented the recruitment of monocytes into the alveolar space.

## Discussion

**Fig. 7:**
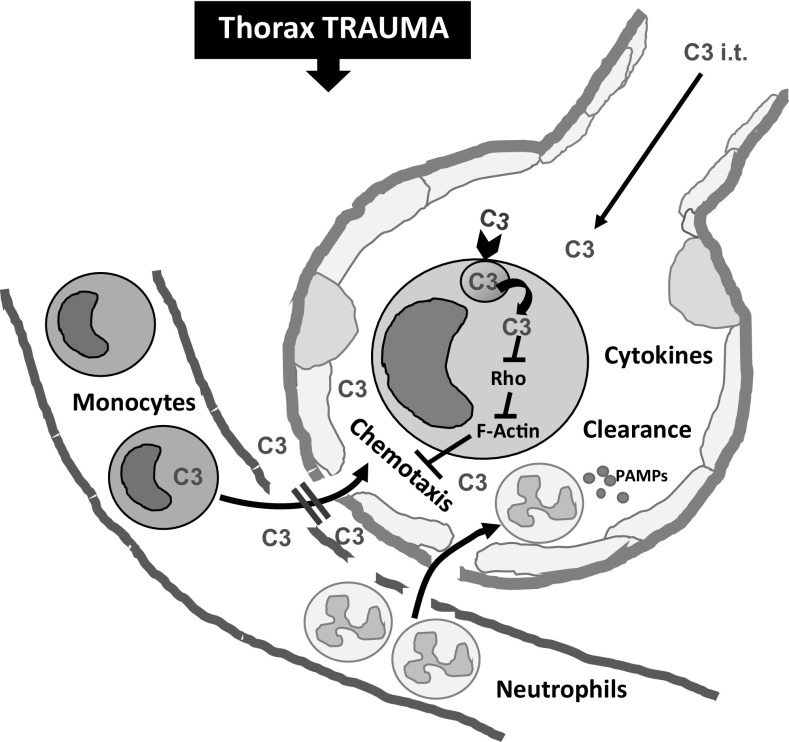
Schematic representation of the inhibitory effect of the Rho-inhibiting C2IN-C3lim fusion toxin on the chemotactic recruitment of monocytic cells from the blood into the alveolar space after blunt chest trauma (TXT). After blunt chest trauma (thorax trauma), the blood–air barrier between the capillary blood and the alveolar space is partially damaged and neutrophils as well as monocytes are recruited into the alveolar space by chemotaxis. There, these cells release cytokines, which contribute to the local and systemic inflammation and might worsen the outcome of multi-trauma patients. According to the current model, the intratracheally (i.t.) applied Rho-inhibitor C2IN-C3lim (indicated as “C3”) enters the cytosol of monocytes, most likely via receptor-mediated endocytosis and subsequent translocation from endosomes into the cytosol. In the cytosol of monocytes, the C3-catalyzed Rho-ADP-ribosylation inhibits Rho-mediated signal transduction, which interferes with F-actin structure and dynamics thereby inhibiting the chemotactic recruitment of monocytes into the alveolar space

Monocytic cells are an essential part of the innate immune system. Normally, they circulate in the bloodstream and can enter the tissues of damaged or inflamed organs where they differentiate into macrophages which are professional phagocytic cells. Thus, they play a central role for the elimination of damage-associated molecular patterns (DAMPS) and pathogens. After infection or injury, recruited monocytes may release inflammatory cytokines at the site of tissue damage (Srivastava et al. 2005; Seitz et al. 2010). The enhanced monocyte recruitment to injured tissues/organs is triggered by a chemical stimulus and the extravasation across the wall of the blood vessels includes a highly sophisticated sequence of steps such as damage/pathogen sensing, tight adhesion, and dynamic adaptation of cell shape during trans-endothelial migration (Worthylake et al. 2001). However, an accumulation of monocytes is not always beneficial. For example, the chemotactic recruitment of leukocytes including monocytes after blunt chest trauma results in local and systemic inflammation (Seitz et al. 2010; Niesler et al. 2014) (see Fig. 7) which hinders the healing of bone fractures and worsens the outcome of multi-trauma patients (Claes et al. 2017). Therefore, pharmacological strategies for the targeted down-modulation of (extensive) monocyte recruitment might beneficially modulate the innate immune response after severe tissue injury and during systemic inflammatory processes.

In monocytes and macrophages, RhoA and -B are key regulators of the actin cytoskeleton and, therefore, represent attractive target molecules to achieve an inhibition of actin dynamics and thereby a down-modulation of monocyte/macrophage migration (Etienne-Manneville and Hall 2002; Caron and Hall 1998; Nobes and Hall 1993; Chardin et al. 1989; Laudanna et al. 1996; Worthylake et al. 2001; Liu et al. 2002). The clostridial C3 toxins, which mono-ADP-ribosylate RhoA, -B and -C, are the only known specific inhibitors for these Rho GTPases (Aktories and Frevert 1987; for review see Vogelsgesang et al. 2007), and C3bot1 was used in previous studies to inhibit the migration of cultured macrophages in vitro. Here we performed a series of in vitro, ex vivo, and in vivo experiments to demonstrate that the targeted pharmacological inhibition of Rho activity by C3 toxins inhibits the migration and chemotaxis of primary monocytes. Here, we showed for the first time the inhibitory effects of C3 toxins on migration of primary human monocytes in a 3-dimensional model of extravasation ex vivo and found that C3-treatment significantly reduced the chemotactic recruitment of monocytes into the lungs of mice after blunt chest trauma. Moreover, we identified the recombinant C2IN-C3lim fusion toxin, which highly selectively enters monocytes/macrophages and changes their morphology without affecting other cell types such as epithelial cells or fibroblasts (Barth et al. 1998, 1999), as the most efficient toxin among the C3 proteins. This finding is consistent with our earlier observation that C2IN-C3lim was more efficient than C3bot1 or C3lim regarding the intoxication of macrophages of the RAW cell line. C2IN-C3lim also inhibited more efficiently the differentiation of these macrophages to osteoclast-like cells as well as the resorption activity of already differentiated osteoclasts in vitro (Tautzenberger et al. 2013; Gacanin et al. 2017). Most likely, the higher biological activity of this fusion toxin is due to its C2IN domain which is the translocation domain of the *C. botulinum* C2I protein (Barth et al. 1998, 1999). The C2IN might enhance the intracellular trans-membrane transport of C2IN-C3lim across endosomal membranes into the host cell cytosol.

Moreover, the mode of action of C2IN-C3lim on cultured macrophages was transient and after removal of toxin from the medium, the cells recovered from the intoxication, which was not observed for the wild-type C3 toxins from clostridia. We reported earlier that C2IN-C3lim becomes degraded in the cytosol of other mammalian cell types and that cells recover due to de-novo synthesis of RhoA (Barth et al. 1999). However, this reversible mode of action of C2IN-C3lim might be an advantage for its use as an inhibitor of monocyte recruitment to organs in vivo because it should allow for a better controlled mode of action by a repeated application of C2IN-C3lim. In case of over-dosing, the effect can be stopped when no more C2IN-C3lim protein is applied. Taken all together, we believe that C2IN-C3lim represents the best C3 Rho inhibitor among the available C3 toxins for the targeted pharmacological down-modulation of an enhanced monocyte recruitment, e.g. into the damaged tissues after trauma. However, having provided proof-of-concept in this study, for further in vivo studies, the production process of C2IN-C3lim must certainly be optimized to yield sufficient amounts of this protein in good manufacturing practices (GMP)-grade quality. Moreover, since we recently provided proof-of-concept for the spatiotemporally controlled release of purified C2IN-C3lim from a biocompatible protein-DNA hybrid hydrogel to down-modulate osteoclast activity (Gacanin et al. 2017), this approach will be adapted for the targeted application of this Rho inhibiting toxin after trauma.

Although infiltration of neutrophils plays a crucial role in development of acute lung injury (ALI) after trauma, monocyte migration into the lungs seems to significantly add to the pulmonary and systemic immune response. In the blast-induced murine blunt chest trauma model (Knoeferl et al. 2003), various released chemotactic factors such as macrophage inflammatory protein 2 or monocyte chemotactic protein 1 were reported within the alveolar space to lead to recruitment of monocytes into the alveoli (Seitz et al. 2010). In turn, the monocytes released further pro-inflammatory mediators but also supported clearance of apoptotic neutrophils and tissue debris (Niesler et al. 2014). In the setting of burn injury, monocytes/alveolar macrophages were shown to fully mount the respiratory stress response and thereby to potentially damage surrounding tissues. Contemporary, these cells were incapable of clearing a secondary infection with *Pseudomonas aeruginosa* and thereby promoted spreading of the infection to remote tissues and the development of sepsis (Davis et al. 2004). However, in the setting of trauma, specific inhibition of monocyte migration via C2IN-C3lim (Fig. 7) may not only inhibit aggravation of acute traumatic lung and remote organ injury, but also may impair clearance of DAMPS and PAMPS. Preclinical studies are essentially needed to investigate the correct timing and indications. Nevertheless, C2IN-C3lim holds great promise since other clinical entities might greatly benefit from this therapeutic approach, e.g. by inhibition of pulmonary intravascular monocytes which as a “passenger leukocyte population” seem to play a major role in the development of lung transplant-associated injury (Tatham et al. 2017).

## Electronic supplementary material

Below is the link to the electronic supplementary material.

**Movie S1 A + B.** Migration of human monocytes on the two-dimensional surface of the left chamber (ceiling of the chamber is much higher than the diameter of the cells) into the confinement of three-dimensional microchannels. Chemotaxis is driven by 100 nM fMLP in the right chamber. The chambers are connected by seven 5 x 5 µm wide and 100 µm long microchannels. Pictures were taken at a framerate of 0.2/sec. Cell tracking was applied to time lapse movies to calculate the migration speed in the left chamber. **(A)** Control experiment with untreated monocytes. Supplementary material 1 (MP4 5546 kb)

**Movie S1 A + B.** Migration of human monocytes on the two-dimensional surface of the left chamber (ceiling of the chamber is much higher than the diameter of the cells) into the confinement of three-dimensional microchannels. Chemotaxis is driven by 100 nM fMLP in the right chamber. The chambers are connected by seven 5 x 5 µm wide and 100 µm long microchannels. Pictures were taken at a framerate of 0.2/sec. Cell tracking was applied to time lapse movies to calculate the migration speed in the left chamber. **(B)** Inhibited monocyte migration after incubation with C2IN-C3lim (2 µg/mL) for 1 h. Supplementary material 1 (MP4 9856 kb)
